# Evaluation of Psychiatric Consultation Requests in A City Hospital in Turkey: A Retrospective Descriptive Study (2021–2023)

**DOI:** 10.1192/j.eurpsy.2026.11045

**Published:** 2026-07-31

**Authors:** B. Ozturk, Z. Çelik, B. Uslu Terzioğlu, A. Kurtulmuş Çalış, F. B. Parlakkaya Yıldız

**Affiliations:** psychiatry, medeniyet university, istanbul, Türkiye

## Abstract

**Introduction:**

Psychiatric comorbidities are frequent in hospitalized patients, affecting outcomes and increasing costs. Around 30–60% present with disorders such as depression, anxiety, or substance use (1). These conditions prolong hospital stays, worsen symptoms, and reduce treatment adherence (1). Consultation-liaison psychiatry (CLP) enables timely intervention, collaborative care, and improved recovery while lowering economic burden . Although effective internationally, large-scale studies in Turkey are still limited, making evaluation of referral reasons and outcomes essential.

**References:**

**1:** Jansen L, van Schijndel M, J. *Health-economic outcomes in hospital patients with medical-psychiatric comorbidity: A systematic review and meta-analysis.*

**Objectives:**

This study evaluated psychiatric consultations at Göztepe Prof. Dr. Süleyman Yalçın City Hospital (2021–2023). It aimed to describe patient demographics, identify referring departments and reasons for referral, assess psychiatric diagnoses, and examine links between patient characteristics, referrals, and outcomes to guide service optimization.

**Methods:**

This retrospective, descriptive, cross-sectional study was conducted at Göztepe Prof. Dr. Süleyman Yalçın City Hospital. Records of patients aged ≥18 years who were hospitalized or admitted to the emergency department and referred for psychiatric consultation between January 1, 2021, and December 31, 2023, were reviewed. Of 6,456 consultations, 4,368 cases remained after excluding repeats and outpatient referrals (except from the emergency department). Data on demographics, clinical variables, referral reasons, referring services, psychiatric history, and outcomes were collected using a structured form. Diagnoses were made according to DSM-5, and data were anonymized. Descriptive statistics were applied.

**Results:**

Of 6,456 consultations, 4,368 were analyzed (mean age 54.5; 53.7% female). Depression (17.4%) and delirium (14.7%) were most frequent; 17.2% had no psychopathology. Referrals were mainly for suicide/self-harm (18.2%) and agitation (16.2%), mostly from Emergency (28.4%) and Internal Medicine (27.6%). Outpatient care was advised in 89.4%, while 10.6% required admission, largely for suicide risk. Among 794 suicide-related cases, 20.9% had ideation and 56.7% overdosed; other methods were less common.

**Conclusions:**

This study describes psychiatric consultations in Turkey, showing depression, delirium, and suicide-related referrals as most common. Consultations mainly came from emergency and internal medicine, highlighting the need for early evaluation. The findings emphasize CLP’s role in improving outcomes and the importance of stronger suicide prevention and multidisciplinary care.

**Disclosure of Interest:**

None Declared

